# Barley Protein Properties, Extraction and Applications, with a Focus on Brewers’ Spent Grain Protein

**DOI:** 10.3390/foods10061389

**Published:** 2021-06-16

**Authors:** Alice Jaeger, Emanuele Zannini, Aylin W. Sahin, Elke K. Arendt

**Affiliations:** 1 School of Food and Nutritional Science, University College Cork, T12 K8AF Cork, Ireland; 116458826@umail.ucc.ie (A.J.); e.zannini@ucc.ie (E.Z.); aylin.sahin@ucc.ie (A.W.S.); 2 APC Microbiome Institute, University College Cork, T12 K8AF Cork, Ireland

**Keywords:** brewers’ spent grain, barley protein, by-product valorisation, brewing waste, food ingredient

## Abstract

Barley is the most commonly used grain in the brewing industry for the production of beer-type beverages. This review will explore the extraction and application of proteins from barley, particularly those from brewers’ spent grain, as well as describing the variety of proteins present. As brewers’ spent grain is the most voluminous by-product of the brewing industry, the valorisation and utilisation of spent grain protein is of great interest in terms of sustainability, although at present, BSG is mainly sold cheaply for use in animal feed formulations. There is an ongoing global effort to minimise processing waste and increase up-cycling of processing side-streams. However, sustainability in the brewing industry is complex, with an innate need for a large volume of resources such as water and energy. In addition to this, large volumes of a by-product are produced at nearly every step of the process. The extraction and characterisation of proteins from BSG is of great interest due to the high protein quality and the potential for a wide variety of applications, including foods for human consumption such as bread, biscuits and snack-type products.

## 1. Introduction

Barley is the most commonly used grain in the brewing industry for the production of beer-type beverages. Both raw barley and malted barley are used, often in combination with adjunct grains such as rice and corn. Arguably, the most important fraction of the barley grain is the endosperm, comprised of starch granules suspended in a protein matrix, as it provides the growing plant embryo with all the products needed to begin growth. In this review, the proteins of the barley grain will be the focus. In particular, the extraction and application of barley proteins from brewers’ spent grains will be explored. As brewers’ spent grain is the most voluminous by-product of the brewing industry, the valorisation and utilisation of spent grain protein is of great interest in terms of sustainability. There is an ongoing global effort to minimise processing waste and increase up-cycling of processing side-streams in order to support sustainable growth in the coming decades. However, sustainability in the brewing industry is complex. There is an innate need for a large volume of resources such as water and energy, as well as a disconnect between raw materials and the processing facility resulting in an increased carbon footprint [1]. In addition to this, large volumes of by-product are produced at nearly every step of the process including spent grain, spent yeast, wastewater and spent hops, among others. The brewing industry is responsible for a huge volume of waste with ~10,000 tonnes of liquid waste and 137–173 tonnes of solid waste produced per 1000 tonnes of beer [2], so the potential valorisation of these waste products is of great economic interest. Due to its high nutritional value, particularly in terms of high-quality protein, the application of BSG proteins in human nutrition is of particular interest. The addition of BSG has already been examined in breads, biscuits and other bakery products as well snack-type products. In addition to this, applications of BSG include use in animal feed formulation for a range of species [1,2,3,4] as well as use as a potential substrate for other industrial processes, namely biogas production, polysaccharide extraction and phenolic compound isolation [4,5,6].

## 2. Barley Structure

**Figure 1:**
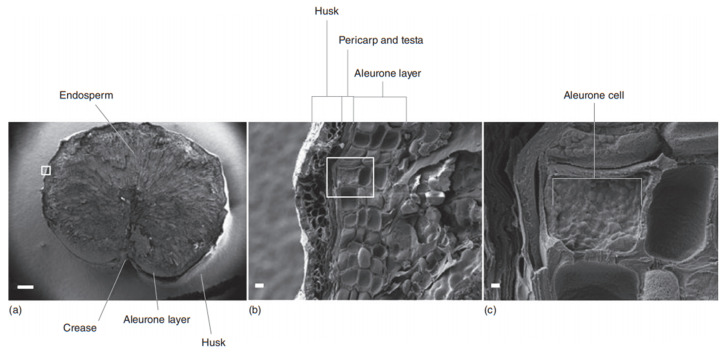
Structure of the barley kernel sectioned transversely (**a**) with detailed husk (**b**) and starchy endosperm (**c**), Reprinted with permission from ref. [9]. Copyright 2013 Elsevier.

Barley (*Hordeum vulgare, vulgare* L.) is a widely cultivated and easily adaptable crop, most commonly used as a raw material for the brewing process. The main components of the barley grain are the embryo, the aleurone layer, the endosperm and the husk (grain covering) (Figure 1). The embryo is the most important living tissue in the grain and develops into the plant. It contains a limited amount of starch, lipids and protein to sustain the embryo prior to germination [7]. The aleurone layer is two to three cells thick and encases the endosperm. It is a living tissue containing protein, phytin, phospholipids, RNA and some carbohydrate and plays a critical role in the regulation of endosperm degrading enzymes [7,8,9]. The grain covering acts to protect the integrity of the grain throughout harvesting and processing. The grain covering can further be divided into the seed coat, pericarp and husk [10]. The husk consists of mainly cellulose and a small amount of polyphenols and bitter substances, whereas the pericarp develops from the ovary wall and acts as a protective cover over the kernel [9]. The husk has proven to aid the filtration process during the lautering step of brewing as well as protecting the grain during malting [7]. However, husk-less cultivars have been shown to be effective in alcohol production, although with an altered malting process [11].

The endosperm consists of starch granules suspended in a protein matrix. The starch is the most abundant component, comprising approximately 60% of grain weight. The endosperm cell walls consist of mainly non-starch polysaccharides, namely β-glucans and arabinoxylans [7].

The barley grain is reported to contain between 8 and 30% protein as a percentage of total mass [7,12,13,14]. This protein is synthesized in the endosperm and the aleurone layer during grain development and accumulates during grain filling. The crude protein content is used to predict malting and, therefore, brewing quality [12,15]. The protein content of the barley grain has a complex relationship with quality with regards to malting barley. While high protein barley would be desirable for feed applications, a lower level is desirable for malting varieties. The ideal protein content for malting barley resides between 10 and 12%, and too high or low a protein content can negatively affect malting quality and, therefore, brewing capacity. In general, a high protein content is undesirable, as there is a strong correlation between a high protein content and a low carbohydrate content, leading to a low extract yield. This may also lead to excessive enzyme activity. However, too low a protein level may lead to insufficient amino acids for the yeast nutrition during the brewing process as well as low enzymatic activity, leading to a lower level of fermentable sugars and, therefore, a poor extract yield [7,15,16,17].

The protein content of the barley grain is highly variable and can be affected by barley cultivar as well as environmental conditions and the addition of fertilizers. In hot, dry and nitrogen-rich environments, it has been shown that the protein content of the barley grain is increased, bringing the protein level above that which is suitable for malting (approx. 11.5% protein) [16,17,18,19]. Increased temperature and reduced rainfall have been shown to have a negative correlation with malting quality as measured by malt extract [15]. However, very high levels of precipitation are also unfavourable, leading to reduced endosperm development [18]. This is undesirable for brewers, as an increased amount of malt will need to be used to produce the beer. Therefore, the development of new barley cultivars to produce grain with a high malt extract and diastatic power while keeping an acceptable protein level is of great interest. Diastatic power refers to the combined activity of α- and β-amylases and β-glucanases. These enzymes play a crucial role in the brewing process, degrading malt carbohydrates to fermentable sugars [17,20].

The application of a nitrogen (N) fertilizer also has a significant effect on barley protein levels. Nitrogen addition was observed to be raising grain protein concentration and reducing malt extract, with a much more significant effect in warmer and drier regions [16]. However, the use of low protein barley genotypes may allow for barley with an acceptable grain protein level to be grown on land with increased nitrates (NO_3_). It has been shown that, while a low-protein genotype is generally associated with reduced diastatic power, the addition of nitrogen fertilizer can increase this level to be similar to that of the most commonly grown malting barley cultivars [17]. The genotype, growing climate, soil composition and fertilizer addition can be of great importance, influencing the performance and quality of the barley malt during the brewing process.

Genetic modification has also been considered for the development of the ideal barley cultivar. While this method does show potential, it cannot yet compete with the more broadly used cultivars in terms of yield [21].

## 3. Barley Proteins

### 3.1. Hordeins/Prolamins

The majority (30–50%) of the barley protein fraction consists of hordeins, a protein belonging to the prolamin group, so named due to their high content of glutamine and proline [14,15,22]. These hordeins (or prolamins) are not single proteins but complex polymorphic mixtures of polypeptides [23]. The other barley proteins consist of a mix of albumins, globulins and glutelin [7]. The hordeins can be further divided into subsections based on their amino acid compositions and extractability characteristics [12,24]. B-hordein is the largest fraction accounting for between 70 and 90% of the total hordein content, and it is the major storage protein of the barley grain. B-hordein can be further broken down into subunits B1, B2 and B3 based on their electrophoretic capabilities. Other fractions include C-, D- and γ-hordeins, with C-hordeins accounting for 10–20%, and the remaining types making up less than 5% collectively [12]. B-hordeins and γ-hordeins are sulphur-rich, while C-hordeins are sulphur-poor. D-hordeins are distinguished by their high molecular weight (>100 kDa) [24]. These differing proteins are coded by specific genes: Hor2 (B-hordeins), Hor1 (C-hordeins), Hor3 (D-hordeins) and Hor5 (γ-hordeins [24]. A study by Howard et al., 1996, observed that the measurement of D-hordein levels in the barley grain gave a more significant single measurement for malting quality spanning a range of climatic and agronomic conditions, compared to total grain protein [15].

### 3.2. Glutelin

Glutelin is the second most abundant fraction in barley storage proteins, making up 35–45% of the total storage protein. It contains high levels of glutamine, proline and glycine, while also being rich in other hydrophobic amino acids [25]. In a study by Wang et al. (2010), barley glutelin was found to have very good emulsifying properties [26]. This is theorised to be due to a balanced ratio of polar and non-polar amino acids, allowing the protein to adsorb easily onto the surface of oil droplets and quickly lower the interfacial tension. However, due to poor solubility, glutelin requires dehydration at pH 11 and further pH adjustment for the emulsification properties to be active, which is not practical for applications in food systems. Deamidation has been found to increase barley glutelin solubility and emulsifying properties [25].

### 3.3. Protein Z

Protein Z is the major barley albumin with a molecular mass of 40 kDa and a pI ranging from 5.5 to 5.8 [27]. Protein Z is a member of the serpin protein group and represents about 5% of total barley albumin. However, protein Z is heat stable and resistant to enzymatic degradation, meaning it survives the brewing process unmodified and is one of the major proteins still present in the finished beer, with a particular role regarding beer foam stability alongside lipid transfer proteins [27,28]. The relationship between the concentration of protein Z and foaming properties has been studied in a few separate instances. It was found that while protein Z is a family of proteins, protein Z4 was the predominant type, as it made up 80% of the protein Z fraction [28]. It was also determined that protein Z plays an integral role in foam stability, as the addition of purified protein Z enhanced foam stability. Similarly, Ayashi et al. (2008) found that protein Z4 and lipid transfer protein 1 (LTP-1) were beneficial to foaming capacity and stability. It is theorised that LTP-1 does this by binding lipids, which are known to negatively influence foam stability [29], while protein Z4 interacts with the α-acids derived from the hops addition. While both the albumin and hordein fractions of the barley protein are capable of foaming, the albumin fraction has significantly higher foam stability. However, denaturation of these protein fractions causes an increase in hydrophobicity, therefore enhancing foaming properties, with albumins being more stable than hordeins. The foam stability of both fractions is improved by exposure to the bitter α-acids derived from the hops, but both protein fractions are also susceptible to hydrolysis by yeast-derived proteinase A, resulting in diminished foam stability [30].

A study by Boba (2010) examined the use of protein Z as a method for monitoring the malting progress of barley grain via glycation. The proteins in barley malt are glycated by D-glucose, a product of starch degradation during malting, and potentially improve foam stability and prevent protein precipitation during wort boiling [31]. It is the lysine and arginine residues that are glycated, with 16% of the lysine residues in protein Z being modified during the Maillard reactions of the malting process. Prolonging of malting increases the level of glycated peptides. Therefore, measuring its concentration using gel electrophoresis, liquid chromatography and mass spectrometry can be used as an efficient measurement of malting progression [31]. This could be applied effectively to control protein variety and quantity in the final beer.

### 3.4. Effect of Brewing Processes on Barley Proteins

The brewing process significantly alters the proteins of the grains. An understanding of the changes to barley proteins that occur during malting and mashing is key to determining how these processes affect the final protein composition of BSG as well as the residual beer proteins.

#### 3.4.1. Malting

Malting is the controlled germination of the barley grain. During malting, partial degradation of the cell walls, starchy endosperm and storage protein occurs [32,33]. The hydrolytic enzymes responsible for this process are produced by the grain during the first stages of germination. For protein degradation, endoproteases and carboxypeptidases are produced and secreted into the endosperm. The endoproteases act to break down the proteins into peptides, while the carboxypeptidases further degrade the peptides into free amino acids [33]. Greater than 70% of the proteins, mainly hordeins and glutelin, in the barley grain are degraded by endoproteases to create smaller peptides during the malting process [33]. A study by Celus et al. (2006) demonstrated that B- and D-hordeins are the main fractions degraded [32]. Adequate modification of the grain constituents during malting is key to the final quality of the beer. Insufficient modification can cause issues such as low extract yields and low fermentability [33].

#### 3.4.2. Starch Degradation Inhibition

Insufficient protein degradation during malting has been shown to inhibit the degradation of starch during the mashing process. A study by Slack et al. (1979) examined this phenomenon and determined that hordein proteins are very closely associated with the surface of the starch granules and can form a protective layer around the granule. Due to this, starch degrading enzymes (namely α-amylase) have limited access to the starch granules, therefore limiting the production of fermentable sugars. It was shown that this effect is even more pronounced for the small starch granules in comparison to the large starch granules. During mashing at approx. 65 °C, the larger starch granules gelatinise, therefore becoming more available to the α-amylase to be converted to maltose. Small starch granules have a higher gelatinisation temperature, and so, the same effect is not observed [34]. During malting, the B- and C-hordeins on the starch granule surface should be largely degraded, allowing for the starch degrading enzymes to easily access the granule. However, in cases where the malt is under-modified or raw adjuncts are introduced, the inhibitory effects of hordeins can be observed. A study by Yu et al. (2018) investigated the same effect and confirmed that barley proteins, in particular the soluble component, play a role in slowing starch degradation. This study also looked at alternate methods by which this occurs [35]. As well as the physical barrier as described by Slack et al. (1979), it is also theorised that the digestive enzyme (α-amylase) binds to the hordein and glutelin and is, therefore, unable to act on the substrate. It is thought that proteins adhere to the starch granules via disulphide linkages, as proteins can be removed by the addition of cysteine [34].

#### 3.4.3. Mashing

Mashing is the first step in the brewing process and continues the enzymatic degradation that began during the malting of the barley grains. Most endoproteases (approx. 90%) will survive the kilning process and will, therefore, be present in the mashing phase [33]. Therefore, protein degradation into peptides and amino acids will continue during mashing. A study by Jones and Marinac (2002) investigated the effect of mashing on malt proteinase activity. It found that the endoproteases maintained a high activity level during the protein rest stage of mashing, where the mash is held at approx. 38 °C to aid protein degradation. However, protease activity rapidly deteriorates when the temperature is raised to 70 °C. This rise in temperature is to facilitate the hydrolysis of malt and adjunct starches to sugars, meanwhile deactivating the proteases. Due to this, soluble protein levels in the wort can be manipulated by the brewer, if so desired, by increasing or decreasing the length of the protein rest phase. It is also noted that attempting to change the ratio of protein fractions within the soluble protein is not likely to succeed, as all of the proteinases appear to denature at the same temperature [36]. Due to the near-total deactivation of the proteases during the starch conversion phase, elongation/shortening of this step would have no effect on wort protein levels. During mashing, a significant fraction of grain proteins are degraded and solubilised, and through wort boiling, they are glycated and coagulated to form aggregates that can be separated during wort boiling as ‘hot trub’ [37]. A study by Celus et al. (2006) determined that during mashing, disulphide bonding is induced between B- and C-hordeins. While it was previously thought that B- and D-hordein (and glutelin) were the main components of the aggregate, this study suggests that it is instead composed of mainly B-hordeins, in which some C-hordein is enclosed [32]. These aggregated hordeins have been reported to cause mash separation issues [32,34,35,38]. An earlier study by Moonen et al. (1987) also described the formation of this protein complex during mashing, consisting of residual malt proteins and glutelins. It also examines how this complex negatively impacts the lautering and filtration processes [38]. This once again highlights the importance of sufficient protein degradation during the malting stage, as poor-quality malt with insufficient modification will contain a higher level of residual protein as well as a lower enzyme activity, therefore causing more issues regarding gelation.

This impenetrable protein complex persists as a coating on the residual spent grains, the upper section of the ‘oberteig’ layer. Therefore, they are an important fraction of the brewers’ spent grain (BSG) protein complement. Due to the impenetrable nature of the matrix, protein extraction may need to involve pre-treatments and/or vigorous disruption procedures [39].

The methods behind the formation of these aggregates are largely unknown. Moonen et al. (1987) hypothesised that high-molecular-weight subunits (such as D-hordeins) form the ‘backbone’ structure of these gels. This idea is supported by Skerritt and Janes (1992), who used protein assays, electrophoresis and HPLC to examine the relationship between these disulphide-linked protein aggregates and malting quality. It was determined that D-hordeins were the slowest to extract and are, therefore, assumed to act as the ‘backbone’ of the gel matrix [40]. An increased level of sodium dodecyl sulphate (SDS) unextractable proteins, namely B1- and B2-hordeins, indicates a lower-quality malt, as hordein subunits are more readily and easily extracted from good-quality malt [40].

#### 3.4.4. Residual Beer Proteins

Many studies have focused on comparing the protein profiles of malts with the protein profiles of the final beer product. One such study by Klose et al. (2010) examined the protein profiles of the malt and beer as well as the intermediate stages (wort and hot trub) using two-dimensional gel electrophoresis as well as lab-on-a-chip technology. It was determined that the 500 mg of proteinaceous material present [41] in the final beer originated from the initial grain, where increased inherent levels of cysteine and/or lysine allowed the proteins to be resistant to enzymatic degradation and increased their heat stability [27]. This need for heat stability was also noted by Curioni et al. (1995), where the major fraction of residual protein in beer was determined to comprise of two polypeptides with an approximate molecular weight of 40 kDa, later determined to be two albumins coinciding with the characteristics of protein Z [41]. It was also noted that upon examination of the precipitated protein in the ‘hot-trub’, the molecular mass of the peptides was low, indicating that the enzymatic degradation of proteins established during malting continued throughout mashing [27].

## 4. Overview of BSG

**Figure 2:**
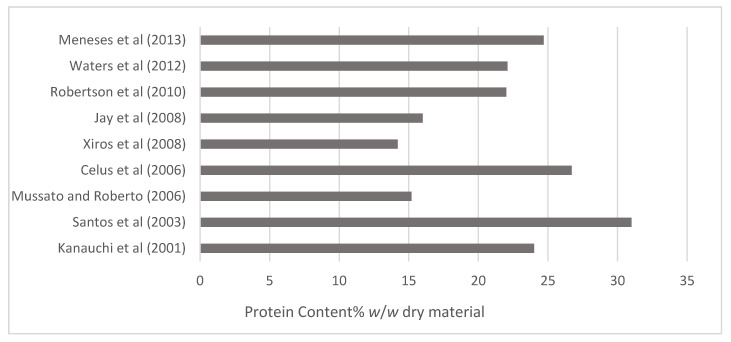
Protein content of brewers’ spent grain (BSG), % *w*/*w* dry matter [32,42,43,44,45,46,47,48,49].

Brewers’ spent grain (BSG) is the most abundant by-product of the brewing process, consisting of up to 85% of total brewery waste [6]. On average, 20 kg of wet BSG is produced for every 100 L of finished beer [10]. Due to an ever-increasing interest in waste reduction and by-product valorisation, the fractionation and application of BSG-derived ingredients is an expanding field of study. BSG is what remains of the initial brewing grains after being subjected to the malting and mashing processes. Therefore, it consists of mainly the husk–pericarp–seedcoat, meaning BSG is rich in both cellulosic and non-cellulosic polysaccharides, as well as lignin. While rich in fibre, it is also rich in protein, with the protein fraction accounting for approx. 19–30% of total grain composition as outlined in Figure 2 [32,42,43,44,45,46,47,48,49].

As well as a high protein content, BSG also contains a high level of essential amino acids, as outlined in Table 1. These essential amino acids constitute approx. 30% of the total protein, and lysine is of particular interest, as it is generally the limiting amino acid in cereal foods for human consumption [48]. In a study by Connolly et al. (2013), protein isolates prepared from pale and black BSG were characterised for amino acid composition. Glutamine and proline were the most abundant amino acids present in both isolates, and different temperatures were used during the protein extraction to determine the effect of temperature. The results showed that the pale BSG, extracted at 50 °C, contained the highest levels of all amino acids, except for cysteine [39]. Glutamine, proline and leucine were the most abundant, while the sulphur-containing amino acids, methionine and cysteine, were the scarcest. The isolate extracted at a lower temperature (20 °C) had a lower amino acid content overall. However, it was similar to the 50 °C in that glutamine and proline were the most abundant, while the sulphur-containing amino acids were present in the lowest levels. The black BSG isolates both contained a lower level of amino acids than their pale counterparts. This is most likely due to the roasting of the malt at high temperatures (>200 °C), where amino acids may be degraded or used in Maillard-type reactions. Essential amino acids are crucial for human health and can be lacking in certain foods, therefore requiring fortification. Due to the presence of a high level of essential amino acids, BSG-derived proteins as fortification agents in foodstuffs for human consumption present an economical solution to these issues.

### 4.1. Adjuncts

Adjuncts are ingredients, other than malted barley, used in brewing to provide additional carbohydrates to contribute to sugars in the wort. While the proteins of the barley grain are the focus of this review, if discussing proteins in brewers’ spent grains, it is important to consider that alternative protein sources may also be present.

In certain countries, namely Germany, Switzerland and Greece, the purity law (‘Reinheinsgebot’) states that beer may only be made from water, malt, hops and yeast, so adjuncts are prohibited. Generally, adjuncts are used to reduce raw material cost and/or to add certain desirable qualities to the finished beer [50,51]. While they are predominantly cereal-based, sugar-based syrups can also be used. The most common cereals used as adjuncts are rice and maize, as well as raw, un-malted barley. Due to differing gelatinization temperatures, adjuncts often have to be ‘cooked’ separately from the main mash in a double mash conversion system [52]. However, triticale can be added into the main mash as it has a lower gelatinisation temperature of 59–65 °C, well within normal mashing temperatures [53]. A study by Agu (2002) also investigated the possible use of sorghum as a brewing adjunct alongside barley. It was discovered that sorghum has the potential to release higher levels of peptides than commonly used maize and was very effective at a 5% addition, whereas at 20% addition, a decrease in total wort peptides was noted [54]. A separate study by Glatthar et al. (2005) investigated the use of un-malted triticale as a brewing adjunct and noted that the wheat and rye hybrid held great potential for use as a brewing adjunct. While adjunct addition levels vary widely, high levels of addition can present increased effects on sensory characteristics as well as technological difficulties, such as increased viscosity [51,53,55]. In a study by Yorke et al. (2021), it was found that while 30% adjunct addition had little to no observable effect on beer characteristics, 60% addition presented increased sensory differences, low free amino nitrogen and dramatically altered the fermentation profile [51].

The protein contents of adjuncts can greatly affect wort quality. The balance between adjuncts and malt must be carefully monitored as adjunct addition can ‘dilute’ the enzymatic activity of the malt, therefore requiring a malt of a higher diastatic power or the addition of commercial enzymes [52]. A study carried out by Schnitzenbaumer et al. (2012) investigated the effect of using oats as a replacement for malted barley. It was noted that the replacement of 20% or more of malted barley with oats resulted in a decrease in the amount of free amino acids and total soluble nitrogen, as well as decreased extract and significantly reduced foam stability.

Due to the frequent use of adjuncts in the brewing industry, the analysis, extraction and utilization of brewers’ spent grain protein is not only dealing with barley proteins but also potentially those of adjuncts used in the brewing process.

### 4.2. Rice Protein

Rice is a commonly used adjunct in brewing to produce a light and clean tasting beer. Due to its low cost and high starch content, it is used to supplement the carbohydrates available from the barley and malt, leading to increased fermentative capability. Brewers’ rice is generally produced as a by-product of the edible rice milling industry, as up to 30% of the grains may be fractured and deemed unsuitable for the edible rice market [56]. While the starchy endosperm is the main component of the rice grain (89–94%), brown rice contains 6.6–7.3% protein, milled rice 6.2–6.9% protein, and basmati rice contains 8.2–8.4% protein [57]. Although levels can vary greatly depending on the environment, soil and cultivar, in general, this protein level is lower than that of both barley, barley malt, maize and sorghum [58]. As opposed to the barley grain, where the major proteins are the endosperm-specific prolamin storage proteins, the major protein in the rice grain is a glutelin-type storage protein, containing 63.8–73.4% glutelin. Other proteins present include water-soluble albumins (9.7–14.2%), salt soluble globulins (13.5–18.9%) and alcohol soluble prolamins (3.0–5.4%) [57]. Rice proteins have been shown to be significantly resistant to hydrolysis [59]. As a result, the addition of a rice adjunct provides very little free amino nitrogen (FAN), and this must be compensated for by the barley and malt fractions.

### 4.3. Maize Protein

Alongside rice, maize is another grain extensively used as an adjunct in the production of beers. In a similar way to rice, the high volume of starchy endosperm serves to supplement the sugars provided by the barley and barley malt alone for a more efficient and economical production process. A study by Agu (2002) describes the effects of maize addition at 5–20%. The results showed a decrease in extract recovery but an increase in levels of FAN and peptide nitrogen when compared to barley adjuncts [54]. Maize contains ~10% protein [60]. Similarly to barley, the major proteins present in maize are endosperm-specific prolamin storage proteins. These are mainly small, 19–25 kDa α-prolamins [58].

## 5. Protein Extraction Methods

### 5.1. Innate Protein Extraction Methods

Several methods of protein extraction from the innate barley grain have been explored in the literature to date. Generally, grains are milled and/or pearled before being exposed to an extraction buffer [26,61,62]. Extraction solutions for barley protein fractions include salt, alcohol and alkaline. In a study by Wang et al. (2010), the hordein fraction was isolated by using an ethanol solution (55–75% *v*/*v*), whereas the glutelin fraction was extracted from the residues by application of an alkaline solution (pH 9–11.5). The hordein fraction was isolated using a rotary evaporator to remove ethanol, while the salt and alkali solubilised fractions were adjusted to pH 5 to facilitate protein precipitation [26].

### 5.2. Extraction of Proteins from BSG

In order to utilise each of the valuable components of BSG, methods for separating the fractions must be determined. In order for barley proteins to be successfully commercialised and used in an industrial setting, efficient extraction methods are needed to separate the valuable proteins from the spent grain. Some of the potential protein extraction methods include alkaline extraction, acid extraction and filtration, as well as more novel techniques such as ultrasonic treatment and pulsed electric field treatment.

### 5.3. pH Shift Extraction Methods (Alkaline Extraction)

Alkaline extraction is the most widely used and well-known methods for protein extraction. A study by Cavonius et al. (2015) explains the mechanics of this method well. At an alkaline pH, the proteins in a system obtain a net negative charge, increasing repulsion within and between the protein molecules. The interactions between the protein molecules and water are promoted; therefore, the proteins are solubilised. When the pH is lowered to the proteins isoelectric point (pI), this negative charge is lost and the protein’s interaction with the water is minimized. This leaves the proteins insoluble and allows them to precipitate out of solution [63]. In this study by Cavonius et al. (2015), the ‘pH-shift’ method was applied to microalgal biomass, but the same principle can be applied to BSG.

Alkaline extraction was utilised in a study by Celus et al. (2007) to create a BSG protein concentrate (BPC). A solution of 0.1 M NaOH was used, and the mixture was held at 60 °C for 60 min to allow for optimum extraction. Following this, the proteins were precipitated by adjusting the pH of the filtrate to pH 4.0 using 2.0 M citric acid, and this precipitate was separated using centrifugation. The resulting BPC contained 60% protein on a dry weight basis [64].

A more recent study by Connolly et al. (2013) explored and characterised the protein-rich isolates from wet pale and wet black brewers’ spent grain using alkaline extraction. While KOH and Na_2_CO_3_ were considered for use, NaOH was determined to be the most effective. It was determined that 110 mM was the most effective and efficient concentration of NaOH for extraction, even though extracted protein peaked at 200 mM. Several other parameters for optimal protein extraction were also explored in this study. Generally, protein extraction for both types of BSG increased over a range from 20–60 °C with pale BSG increasing from 37.17 to 88.20 mg g^−1^ and black BSG increasing from 37.02 to 64.32 mg g^−1^ [39]. Temperatures over 60 °C were not considered due to the risk of protein denaturation. This is in agreement with previous studies by Bals et al. [65] and Celus et al. [64]. The optimum weight/volume ratio was also determined to be 1:20. The barley proteins present in the alkaline extracts of BSG consisted mainly of hordeins.

A study by Diptee et al. (1989) explored the various parameters involved in protein extraction yield from brewers’ spent grain using response surface methodology. These variables included time, temperature and particle (grain) size. While the efficacy of commonly used protein extraction solutions on BSG proteins has been debated, the extraction solution used in this study consisted of 3% sodium dodecyl sulphate and 0.6% Na_2_HPO_4,_ followed by precipitation of the protein in ethanol. Using this method, a 60% protein yield was obtained using a BSG: extractant ratio of 2.5:100 and by heating the mixture to 90 °C for 95 min [66]. This was in agreement with the maximum value predicted using response surface methodology.

As seen through extensive use, alkaline extraction is a viable and efficient method to extract proteins, namely hordeins and glutelin, from BSG. The protein obtained from BSG could potentially be used in a variety of applications, including the food and nutraceutical industries.

### 5.4. Filtration

Filtration, including microfiltration and ultrafiltration, has been a commonly employed method of separating out fractions based on molecular size. Examples include the use of membrane separation to separate the solid fractions of corn, resulting in high protein corn gluten meal (67% protein), high-fat corn germ, corn starch and high fructose corn syrups [67].

A study by Tang et al. (2009) applied ultrafiltration to brewers’ spent grain as a method of protein extraction. A BSG extract was prepared using ultrasound-assisted extraction with a sodium carbonate buffer, then filtered through a nylon cloth and centrifuged. The resulting mixture was used as the feed solution for ultrafiltration. Protein recovery by ultrafiltration was highly successful, with more than 92% of the protein being retained using both 5 and 30 kDa membranes [68]. The protein contents of the final products were 20.09% protein when concentrated using the 5 kDa membrane and 15.98% protein when concentrated using the 30 kDa membrane [68]. Connolly et al. [69] also used membranes of 10 and 30 kDa to concentrate proteins from a BSG preparation. A benefit of using this type of fractionation is the lack of added heat, yielding a higher-quality protein product.

### 5.5. Pre-Treatments

The effects of various pre-treatments of the BSG have also been widely investigated.

Shearing as a pre-treatment proved to be beneficial. It is thought to be due to the physical disruption of cell membranes and lignocellulosic material that allows for easier extraction of proteins. The use of acid as a pre-treatment was also investigated, but in this study, it did not appear to have any effect on protein extractability [39]. Hydrogen peroxide proved to be effective, as its application increased protein yield by 27%. However, this method of pre-treatment is not frequently used due to its potential to cause oxidation of the extracted protein.

A study by Qin et al. (2018) outlines the effects of several differing pre-treatment strategies on the protein extractability of BSG. A combination treatment of alkaline and acid seemed to be effective, with up to 95% protein extraction achieved [70]. However, it was discovered that a high percentage of lignin and carbohydrates were solubilised with the protein, meaning the process was not selective for proteins only. This would limit its practical applications, as a purer protein fraction is usually the most desirable. A single step dilute acid pre-treatment using H_2_SO_4_ was proven to be effective at extracting up to 90% of the BSG protein; however, a similar issue with the carbohydrates and lignin was present. The benefits of hydrothermal pre-treatments were examined at differing solid to liquid ratios, differing temperatures (30 to 135 °C) and increasing time (1 and 24 h). It was found that this allowed for 64–66% protein extraction and was more selective for proteins than the acid and alkaline treatments [70]. Hydrothermal treatment is potentially a very good option for optimisation of protein extraction from BSG, as it requires no addition of chemicals and can be carried out at a relatively low temperature to preserve protein quality but also minimise energy use when compared to acid treatment.

### 5.6. Enzymatic Treatment

The use of enzymes, namely proteases, to separate proteins from BSG has also been explored in literature. In addition to alkaline treatment, enzymatic treatment could optimise protein extraction potential from BSG. A study by Niemi et al. (2013) states that while up to 53% of proteins in BSG can be solubilised in alkaline conditions without protease addition, up to 76% was solubilised with the addition of an alkaline protease, in addition to carbohydrase treatment.

Alcalase, an alkaline protease extracted from *B. licheniformis*, has been reported to be effective in aiding to separate the protein fraction from BSG [64,71,72,73]. Determined to be capable of solubilising and extracting up to 77% of BSG proteins, creating peptides with a much lower molecular weight than other less effective proteases [73]. It was found that Alcalase performed better at pH 8.0, requiring a pH adjustment of the raw materials. To achieve maximum protein solubilisation, 10–20 µL of enzyme per g dry matter was required to work for approx. 4 h. However, significant protein separation, up to 64%, was observed using does as low as 1.2 µL [73]. It was also noted through amino acid analysis that an Alcalase treatment was particularly efficient at solubilising the proline and glutamine components of barley hordeins, more so than other amino acids. A study by Qin et al. (2018) also aimed to utilise the protein solubilisation effects of Alcalase for protein extraction optimisation from BSG. However, the results differed from those reported in the literature, with only 43–50% protein extraction. It was theorised to be due to the pH of the mixture being slightly too low at pH 6.25 after pre-treatment as opposed to pH 6.5, the optimum pH for Alcalase. Due to this, results differed from those reported in the literature. When the treatment was repeated at pH 8.0, a minor increase in protein solubility was observed, though it was still a lower figure than had been reported previously.

A study by Niemi et al. (2013) investigated the combined use of proteases and carbohydrases to maximise protein solubility. Milled BSG was treated with Depol 740 L in a 10% weight/volume mixture for 5 h at 50 °C with constant stirring. This BSG was then treated with different proteases, including Alcalase, ProMod and Acid Protease A [71]. Alcalase was found to be the most effective enzyme at pH 9.5, solubilising 35% of BSG biomass, while the other proteases only solubilised approx. 14%. It was found that enzymatic carbohydrate digestion was beneficial in maximising proteins solubilisation and extraction from BSG, leading to an increased protein yield. It is theorised to be due to the degradation of important cell structures by the carbohydrate degrading enzymes, allowing greater access to the internal cell structures. It was also noted that prolonged exposure to alkaline conditions, in the absence of a protease, also led to a significant amount of protein being solubilised, indicating that prolonged exposure to a high pH decreases cell wall integrity and increases protein solubility [71]. Depol 740 was also used in combination with Alcalase to improve protein solubilisation in a study by Treimo et al. (2008). While it was found to improve the overall solubilisation of the biomass, the increase was largely in the solubilisation of non-proteinaceous material. Overall, treatment with carbohydrate degrading enzymes had only minor effects on protein solubilisation, as opposed to the larger effect observed by Niemi et al. (2013).

A study by Celus et al. (2007) investigated the technological properties of BSG protein hydrolysates as prepared by enzymatic hydrolysis. Commercially available enzymes, including Alcalase, Flavourzyme and Pepsin, were used over a range of times and concentrations to obtain hydrolysates with varying degrees of hydrolysis (DH). BSG protein concentrate was first prepared by alkaline extraction and then subjected to enzymatic hydrolysis. It was determined that enzymatic hydrolysis generally improved the solubility of BSG proteins at lower pH values, leading to increased protein yield across the entire pH range [64]. A significant difference in technological function was also observed between the samples, based on the enzyme used during the hydrolysis. Emulsification and foaming properties decreased with increasing DH when Alcalase or Pepsin was used, as these properties are reliant on peptides with a MW in excess of 14.5 kDa. However, the opposite was observed for hydrolysates prepared with Flavourzyme [64]. Hydrolysis of the BSG proteins greatly improved their technological functionality. Foaming and emulsification properties, which were negligible in the raw BSG, were significantly improved by the hydrolysis treatment.

The use of thermochemical pre-treatment, as well as carbohydrate and protein hydrolysing enzymes on the fractionation of protein and lignin from BSG was investigated in a study by Rommi et al. (2018). Significant protein solubilisation was achieved using an alkaline protease treatment, bringing BSG protein solubilisation from approx. 15% to almost 100%, in agreement with the studies mentioned previously [64,71,72,73]. The steam explosion treatment reduced protein solubility but increased the efficacy of extract separation in the centrifugation step. Lignin and protein from BSG co-extracted and could only be partially separated using acidic precipitation. Due to this, raw BSY may be preferred in the production of BSG protein hydrolysates, with an aim to limit lignin co-extraction [72].

### 5.7. Ultrasonic-Assisted Extraction

Ultrasonic treatment is a more novel method that has been explored as a means of aiding protein extraction from BSG. In a study by Tang et al. (2010), the optimum ultrasound treatment was determined using response surface methodology, regarding three parameters: time, power and solid/liquid ratio. A treatment time of 81.4 min, at an ultrasonic power of 88.2 W/100 mL extractant and a solid/liquid ratio of 2.0 g/100 mL, was used to predict an optimal yield of 104.2 mg/g BSG, and this was in accordance with the experimental value [74]. Ultrasonic treatment of BSY was also determined to improve certain functional characteristics, namely fat absorption capacity, foaming properties and emulsifying properties, as well as improving extraction yield [75]. When ultrasound treatment is used in combination with enzymatic treatment, as in Yu et al. (2019), enzymatic loading and incubation time can be reduced, and protein solubility significantly increases [76].

### 5.8. Pulsed Electric Field

Pulsed electric field technology (PEF) is another novel extraction method for food compounds that is gaining interest. PEF consists of the application of short-duration pulses of an electric current through a sample that is secured between two electrodes [77]. The enhanced extractability of samples post-PEF is due to the dielectric disruption of cell membranes as a result of exposure to an electric current. This technology has already been explored as a non-thermal and, therefore, cheaper and more sustainable method of food preservation and microbial inactivation technique [77,78]. Outside of food preservation and antimicrobial applications, PEF has also been explored as a means of increasing juice yield from alfalfa leaves [79] and also as a pre-treatment to increase the extractability of proteins and phenolic compounds from light and dark BSG extracts [80]. This study by Kumari et al. (2019) showed that PEF assisted extraction significantly increased the level of free amino acids in the light BSG extract, and all essential amino acids were present in both extracts, with tryptophan being the only exception [80]. PEF treatment has also been explored in combination with ultrasound treatment as a method of optimising the extraction of phenolic compounds and proteins from agro-industrial by products [77] and olive kernels [81].

The more well-known methods of protein extraction, namely alkaline extraction and enzymatic treatment, are very suitable for protein extraction from BSG and are supported by large amounts of literature documenting their success. However, there are potential downsides and room for optimization with regards to the amount of solvent and/or enzyme required as well as heat treatments that could negatively impact protein quality. This leaves much room for optimization with a focus on cost, quality and sustainability. The more novel methods have little to no supporting literature with regards to their use for BSG but could benefit from being a ‘cleaner’ and more economical method of protein extraction, as there is no need for reagents, as well as potentially yielding protein of a higher quality due to a lack of thermal treatment. However, it is clear that the best course of action for protein extraction from BSG is a combination of the methods discussed above, as it has been shown in BSG and other food systems that combining methods allows for optimization of protein yield [68,70,72,77,81].

## 6. Applications

### 6.1. Animal Nutrition

Due to its high protein and fibre content, BSG is most often used as a component of animal feed as either a wet or dry feed. In combination with cheap and widely available nitrogen sources (such as urea), BSG can provide all essential amino acids [6]. The introduction of BSG into the diet of milking cattle has been shown to increase milk yield, total milk solids and milk fat when compared to an animal on a control diet. Protein levels and lactose content were not significantly affected by the change in diet [6,82,83,84]. While most commonly used in cattle, the use of BSG in the feed of other animals has also been explored. In a study by Mukasafari et al. (2017) BSG was successfully used to substitute up to 50% of sow and weaner meal without any negative effects on the quality of the pigs [85]. A study by Yaakugh and Tegbe (1994) investigated the replacement of maize in the diet of pigs with dried brewers’ grains. It was found that, with a replacement level of up to 45%, the daily weight gain was significantly depressed, but the final carcass weight was not affected [86]. A study by Oh et al. (1991) investigated the incorporation of 15% treated BSG into the diets of poultry. The treatment consisted of partially hydrolysing the grains by cultivating the fungus *Trichoderma reesei* on the grains to alter the amino acid profile and release soluble sugars. Incorporation of these grains at 8 and 12% into the diets of broiler chicks resulted in a significant improvement in their growth and feed conversion ratio in the first 4 weeks. No further improvements were seen after 6 and 8 weeks of growth [87].

The use of BSG has been investigated as a source of protein in aquafeeds as a sustainable alternative for fishmeal and oil to reduce reliance on marine resources. Partial replacement of fishmeal with BSG (20–30%) in the feeding of rainbow trout and gilthead seabream showed similar results in digestion efficiency when compared to the control, where fishmeal was used as the main protein source. The BSG replacement showed good protein, amino acid and lipid digestibility, making BSG a suitable alternative for fishmeal, increasing the sustainability of both industries [3]. Similarly, San Martin et al. (2020) explored the use of an enzyme hydrolysis process in a bid to increase the digestibility of BSG proteins in aquaculture feeds. While further studies are required into the benefits of hydrolysis, both the hydrolysed and non-hydrolysed BSG proteins have shown good digestibility and would be a suitable fishmeal alternative, improving economic and environmental sustainability [2].

Due to the very high water content of BSG at the point of production (77–81%) [6], the transportation and storage of BSG for use as animal feed presents a challenge in minimizing microbial growth that may cause illness in animals, as well as general material degradation. There are guidelines regarding the preservation of BSG, suggesting the use of preservatives such as benzoate, propionate and sorbate to extend stability [6,10]. However, these measures only work to extend shelf-life for 4–5 days, so a more effective preservation technique is required for longer storage times. Drying is the most commonly employed methods of stabilizing wet BSG by reducing microbial growth. A study by Bartolome et al. (2002), compared three preservation methods: oven drying, freeze drying and freezing. Freezing was deemed to be unsuitable due to the potential for alterations in arabinose content and lack of suitability for large volumes. Oven drying and freeze drying were found to be equally effective in terms of reducing volume and preventing changes to the composition. However, from an economical and cost standpoint, oven drying was determined to be the most effective methods for removing moisture from BSG and stabilizing the product [88].

### 6.2. Bio-Degradable Film

A study by Lee et al. (2015) describes the use of brewers’ spent grain protein (BGP) in the production of bio-degradable composite films. The brewers’ spent grain protein was extracted by alkaline extraction, as described by Celus et al. (2007). It was determined that the addition of chitosan improved the physical and mechanical properties, including elasticity and tensile strength. The optimum levels of BGP and chitosan were determined to be 3 and 2%, respectively. In addition, the chitosan contributed to enhanced antimicrobial and antioxidant properties of the films, inhibiting the growth of *Staphylococcus aureus*, *Listeria monocytogenes*, *Escherichia coli* and *Salmonella typhimurium* [89]. The potential use of brewers’ spent grain protein in biodegradable packaging materials is a very exciting potential use of the by-product.

Barley protein was also used to produce barley protein–gelatine composite films in a study by Song et al. (2012). The physical properties of this film were investigated, and it was found that increasing levels of barley bran protein caused tensile strength and elongation at break value to decrease. However, increasing the proportion of gelatine increased tensile strength but still reduced elongation value. It was determined that the optimal composition for film production was 3 g barley bran protein, 3 g gelatine and 100 mL sorbitol in 100 mL of film-forming solution. Grapefruit seed extract was also incorporated into the film as an antimicrobial agent and proved to be successful in reducing the growth of pathogenic bacteria when used in salmon packaging [90].

### 6.3. Food Applications

The implementation of brewers’ spent yeast in bakery products has been widely studied. While these applications are not always protein-focused, in many cases, the protein fraction of BSG works synergistically with other compounds to improve nutritional and/or technological functionality. BSG addition to foodstuffs can improve protein content significantly but can also drastically increase levels of dietary fibre, which is very desirable for human health [48,91,92].

#### 6.3.1. Biscuits

The use of protein isolated from brewers’ spent grain has the potential for use in bakery products to improve nutritional and functional properties. A study by Zong et al. [93] implemented spent grain protein in cookies and studied the effects of the addition on the sensory and textural characteristics of the product. This addition improved the overall flavour and nutritional value of the cookies [77].

#### 6.3.2. Bread

The use of BSG and fermented BSG in the fortification of bread has been explored by Waters et al. At 10% addition, dough displayed improved handling characteristics. As well as this, softness and staling were improved in both cases. While sweetness was decreased and acidulous flavour increased, both bread types were acceptable at a 10% addition level [48]. This acceptability, as well as the increase in nutritional value with regards to protein, dietary fibre and minerals, makes BSG a very interesting raw material for food product fortification.

The effect of BSG supplementation on bread doughs was also explored. In a study by Ktenioudaki et al. (2013), BSG and apple pomace were added to wheat dough, and their effect on the doughs physiochemical properties studied. The BSG was found to be high in protein (20.8%) and high in dietary fibre (60.5%). The rheological and pasting properties of the dough were greatly altered, mainly thought to be due to the high fibre content. Increasing by-product addition significantly reduced peak viscosity, holding strength, breakdown, final viscosity and setback values, as well as strain hardening index. Meanwhile, biaxial extension viscosity was higher for the supplemented dough and the storage modulus G′′ was increased. These changes all indicate significant structural differences between the un-supplemented and supplemented doughs [94].

#### 6.3.3. Snacks

Dried and milled BSG has been used as a means to increase the protein content of extruded snacks [95]. BSG was incorporated at 10–30% levels in combination with wheat flour, corn starch and other ingredients and extruded using a twin-screw extruder. This addition significantly increased crude protein content, as well as increasing the phytic acid level and bulk density. Meanwhile, sectional expansion and cell area were reduced. A follow-up study examining the effect of altering water feed rates using a combination of BSG and different flours was performed. The total dietary fibre of the wheat flour and BSG (WBSG) and cornflour and BSG (CBSG) mixtures were found to increase significantly. Generally, TDF increased with increasing water feed level for WBSG sample, while the opposite was observed with the CBSG samples [96].

A similar study by Ainsworth et al. (2007) also looked into the effects of BSG addition in the formulation for a chickpea-based extruded snack. It was determined that BSG addition (0–30%) resulted in decreased expansion, which is in agreement with other studies. However, increased screw speed had the opposite effect. The BSG product also displayed increased phytic acid, resistant starch and protein in vitro digestibility [97]. A study by Ktenioudaki, Crofton, et al. (2013) investigated the use of BSG in a crispy snack product as a means to increase fibre content. It was found that a 10% BSG addition almost doubled the fibre content of the product without compromising product acceptability [92]. While often used to increase protein levels in food products, BSG can also enhance other nutritional characteristics such as fibre content.

A study by Singh et al. investigated the potential use of novel drying methods on brewers’ spent grain for use as a plant protein source in baked chips. Vacuum microwave drying (VMD) was proven to be an efficient method for drying BSG, as it reduced drying time, showed a high drying efficacy and a high overall acceptability in the baked snack when compared to those prepared using oven-dried or freeze-dried BSG. The applications of VMD technology are interesting from a sustainability standpoint as well as a nutritional standpoint, as the lack of a high temperature, due to the lowering of the boiling point by the vacuum, results in a higher-quality protein ingredient [98].

#### 6.3.4. Beverages

The research surrounding the inclusion of BSG-derived ingredients in beverage formulation is limited. A study by McCarthy et al. (2013) investigated the use of phenolic compounds extracted from BSG as antioxidants in fruit beverages and determined that BSG extract addition resulted in significantly increased antioxidant activity, as measured by the ferric reducing antioxidant power (FRAP) assay [5]. While other studies incorporating this concept are rare, several patents are available (Table 2) regarding the extraction and use of BSG-derived ingredients in beverage applications.

### 6.4. Barley Protein Hydrolysates

Due to a lack of solubility of barley proteins, functionality in food applications is limited. To improve ingredient functionality, protein hydrolysis can be implemented.

Celus et al. (2007) used enzymatic hydrolysis as a means to potentially improve the solubility, colour, emulsification and foaming properties, as well as cause a change in molecular weight distribution and hydrophobicity of BSG proteins. Differing degrees of hydrolysis were obtained by subjecting the protein concentrate to differing concentrations of three enzymes (Alcalase, Pepsin and Flavourzyme) for varying amounts of time. Generally, hydrolysis of the BSG protein improved emulsion and foaming. This somewhat agrees with a separate study by Yalçın et al. (2008), where barley protein hydrolysates were determined to have slightly improved foaming characteristics when compared to barley protein isolates, but the difference was slight [99]. However, those hydrolysates prepared with Alcalase and Pepsin showed a decrease in these characteristics with an increasing degree of hydrolysis. Characterisation of these hydrolysates showed that a relatively high molecular weight and a high surface hydrophobicity are desirable for enhanced physiochemical properties [64].

Enzymatic hydrolysis of barley proteins can greatly improve antioxidant capabilities and metal-binding activity [100,101,102]. A study by Chanput et al. (2009) examined the antioxidant properties of partially purified proteins from a variety of sources, including barley hordeins. Hydrolysates of these proteins were prepared using an enzymatic treatment of pepsin, followed by trypsin. Antioxidant and reducing properties were investigated, and the partially purified C-hordein displayed a high reducing capacity when compared to the B- and D-hordeins. It was generally found that for all protein hydrolysates, antioxidative and reducing capacities were greatly increased after enzymatic digestion with pepsin and trypsin [101]. A recent study by Ikram et al. (2020) followed on from this idea, investigating the effects of pre-treatments such as ultrasonic and heat treatments on the enzymatic hydrolysis of BSG proteins by Alcalase and the extent to which these pre-treatments, in combination with hydrolysis time, altered the antioxidant activity of the hydrolysates. The treatments selected were an ultrasonic treatment of 40 or 50 kHz and a heating treatment of 50 and 100 °C, while the pre-treatment times were 15, 30 and 60 min. The ultrasonic treatment at 40 and 50 kHz was shown to significantly increase oxygen radical absorption capacity values of the hydrolysates, while the heat treatment at 100 °C greatly increased the ferric reducing antioxidant power (FRAP) assay values [100]. These results indicate that hydrolysed BSG proteins exhibit a higher antioxidative power than untreated material and that these antioxidative capabilities can be further increased with ultrasonic and heat pre-treatments. Therefore, these hydrolysates could prove to be useful food ingredients due to their antioxidant and reducing potential.

Barley hordein hydrolysates also show potential as dietary supplements to enhance mineral bioavailability and solubility [102]. Through enzymatic digestion with Flavourzyme, Alcalase, trypsin and pepsin, barley proteins become hydrolysates with a strong metal ion binding capacity and can significantly increase the solubility of Fe^2+^ Fe^2+^, Ca^2+^, Cu^2+^ and Zn^2+^ [102]. Therefore, BSG proteins could potentially have a useful application in dietary supplements to enhance mineral bioavailability and solubility.

## 7. Conclusions

The increasing interest in sustainability and economising product side streams has led to an increased emphasis on the reuse and valorisation of brewing by-products. As brewers’ spent grain is the most abundant by-product from this industry, the valorisation and utilization of spent grain protein is of great interest, particularly in terms of sustainability. Finding ways to upcycle this cheap and readily available product and apply it in a variety of different settings is a research area that is rapidly gaining traction. BSG is currently extremely underutilised and is mainly used in animal feed formulations due to its low cost and high nutritional value. High levels of essential amino acids in the proteins could be useful in nutritional and functional food applications for human consumption.

To date, BSG has been successfully applied in bakery products such as bread, biscuits and snack-type products, and BSG protein hydrolysates have been found to have increased functionality, including enhanced solubility, foaming and emulsification properties. These hydrolysates have also shown potential to enhance nutrient bioavailability as well as an increased antioxidative and reducing capability. All in all, BSG protein and its hydrolysates have significant valorisation potential, especially with regards to applications in the food industry.

## Figures and Tables

**Table 1:** Amino acid composition of barley, barley malt and BSG—adapted from Waters et al. (2012).

	Barley	Malt	BSG
Total protein (% *w*/*w*)	9.65	8.52	22.13
(% of total)			
Aspartic acid	0.19	0.17	4.81
Glutamic acid	0.85	0.75	16.59
Asparagine	0.23	0.33	1.47
Serine	0.12	0.07	3.77
Glutamine	ND	ND	0.07
Histidine	1.59	1.9	26.27
Glycine	0.08	0.06	1.74
Arginine	0.21	0.23	4.51
Alanine	0.22	0.23	4.12
γ- aminobutyric acid	2.56	0.01	0.26
Tyrosine	0.14	0.14	2.57
Valine	2.56	0.24	4.61
Threonine	0.01	0.02	0.71
Methionine	0.03	ND	ND
Tryptophan	0.01	ND	0.14
Phenylalanine	0.2	0.21	4.64
Isoleucine	0.17	0.17	3.31
Leucine	0.3	0.29	6.12
Lysine	2.52	3.69	14.31

**Table 2:** Patents regarding the use of BSG ingredients in food and beverage products.

Patent Number	Title	Area of Usage	Summary
WO/2018/033521	A process for preparing a beverage or beverage component, beverage or beverage component prepared by such process and use of brewers’ spent grains for preparing such beverage or beverage component.	Food/beverage ingredient	Process of preparing a beverage or beverage component obtained by the fermentation of brewers’ spent grain and a process of preparing such beverage and other foodstuffs.
WO/2018/033522	A process for preparing a beverage or beverage component from brewers’ spent grains.	Food/beverage ingredient	Process of preparing a beverage or beverage component obtained by the enzymatic saccharification and fermentation of brewers’ spent grain and a process of preparing such beverage, as well as other foodstuffs.
WO/2019/034567	A process for microbial stabilization of brewers’ spent grain, microbiologically stabilized brewers’ spent grain and use thereof.	Food/beverage ingredient	Process for treating brewers’ spent grains (BSG) obtained from the brewing process such that the growth of microbes in said grains and subsequent production of microbial toxins are kept below specified levels.
WO/2019/158755	A process for recovering proteinaceous and/or fibrous material from brewers’ spent grains and use thereof.	Food/beverage ingredient	Process of extracting or purifying proteinaceous material and/or fibraceous material from brewers’ spent grain, as well as the use of these materials.

## Data Availability

Not applicable.
